# Mast cell leukemia: a rare case report and literature review

**DOI:** 10.3389/fonc.2025.1537301

**Published:** 2025-02-25

**Authors:** Lu Tian, Xiang Yan, Shaodan Tian, Yang Shen, Qiuyue Fan

**Affiliations:** Department of Oncology and Hematology, Dongzhimen Hospital, Beijing University of Chinese Medicine, Beijing, China

**Keywords:** mast cell leukemia, systemic mastocytosis, rare diseases, clinical case reports, literature review

## Abstract

Mast cell leukemia (MCL) is an extremely rare and aggressive hematologic malignancy, characterized by a poor prognosis and short survival. Currently, no standardized treatment guidelines have been established. This study presents the clinical data of a patient with primary MCL accompanied by C-findings, and analyzes the clinical features, diagnostic challenges, and therapeutic approaches for this disease through a comprehensive review of the relevant literature. Furthermore, the study discusses current perspectives on research developments in MCL. By increasing clinical awareness of MCL, this work aims to provide valuable insights and references for clinicians in the diagnosis and management of this challenging condition.

## 1 Introduction

Mast cell leukemia (MCL) is the rarest subtype of systemic mastocytosis (SM), accounting for less than 1% of all mastocytosis cases. First reported by Joachim in 1906, only four large studies have systematically described MCL to date. The primary characteristic of MCL is the leukemic expansion of mast cells in the bone marrow and other organ systems, resulting in symptoms of mast cell activation that affect multiple organs, including the liver, spleen, peritoneum, and bones (1–3). MCL is associated with an extremely low incidence and high malignancy, with a median overall survival of only 1.6 years (3–5), indicating a poor prognosis. Consequently, more clinical real-world data are needed to improve the understanding of this disease. Here, we present the case of a 50-year-old patient with primary MCL accompanied by C-findings. While the presence of C-findings typically indicates a poor prognosis, this patient has remained stable and free of disease progression for nearly 12 months with symptomatic treatment alone. This case aims to summarize the clinical features of MCL, enhance clinicians’ understanding of the disease, and provide a reference for clinical practice.

## 2 Case report

On June 18, 2024, a 50-year-old male patient with primary MCL presented to the outpatient clinic for the first time. The patient reported experiencing fever and fatigue of unclear origin since February 2024. A complete blood count performed on February 23, 2024, revealed RBC: 2.59×10¹²/L and HGB: 79 g/L, but these findings were not given sufficient attention. By June 2024, the patient’s symptoms of fever and fatigue had worsened, and a repeat blood test on June 18, 2024, showed HGB: 82 g/L. Due to the prolonged fever, fatigue, and anemia, the patient was admitted to the hospital for further evaluation and symptomatic treatment.

### 2.1 Clinical manifestations

The patient presented with fever, fatigue, pruritus, poor appetite, and diarrhea occurring 2–3 times daily, along with noticeable weight loss over a short period. Physical examination revealed mild tenderness in the upper abdomen, splenomegaly, with the spleen’s lower border palpable 8 cm below the costal margin. The patient denied a history of chronic hematologic diseases, a family history of genetic disorders, or any significant occupational or psychosocial exposures.

### 2.2 Laboratory examinations

#### 2.2.1 Hematological tests

Complete Blood Count: WBC: 4.1×10^9^/L, PLT: 153×10^9^/L, RBC: 2.09×10^12^/L, HGB: 69 g/L, HCT: 19.4%, MCHC: 355 g/L, MCV: 93.1 fL. Anemia Panel: TRF: 1.67 g/L, TIBC: 41.7 μmol/L, Fe: 15.4 μmol/L, TS: 36.9%. Vitamin B12 and folic acid levels were normal. Coagulation Tests: PT: 13.7 s, PT%: 72%, APTT: 34.3 s, FIB: 2.77 g/L, TT: 16.4 s, D-Dimer: 366 μg/L, FDP: 2.47 mg/L. Immunological Tests: IgG: 6.9 g/L, C3: 0.648 g/L. Tests for antiphospholipid antibodies, antinuclear antibodies, vasculitis, and tuberculosis antibodies/cell-specific markers were negative. Peripheral Blood Morphology: Erythrocytes: Presence of late normoblasts, anisocytosis, and irregularly shaped red blood cells. Leukocytes: Mast cells constituted 6%. Platelets: Normal in quantity and morphology. Mast cell infiltration was observed, prompting further bone marrow aspiration for definitive diagnosis.

#### 2.2.2 Bone marrow cytomorphology

**Figure 1:**
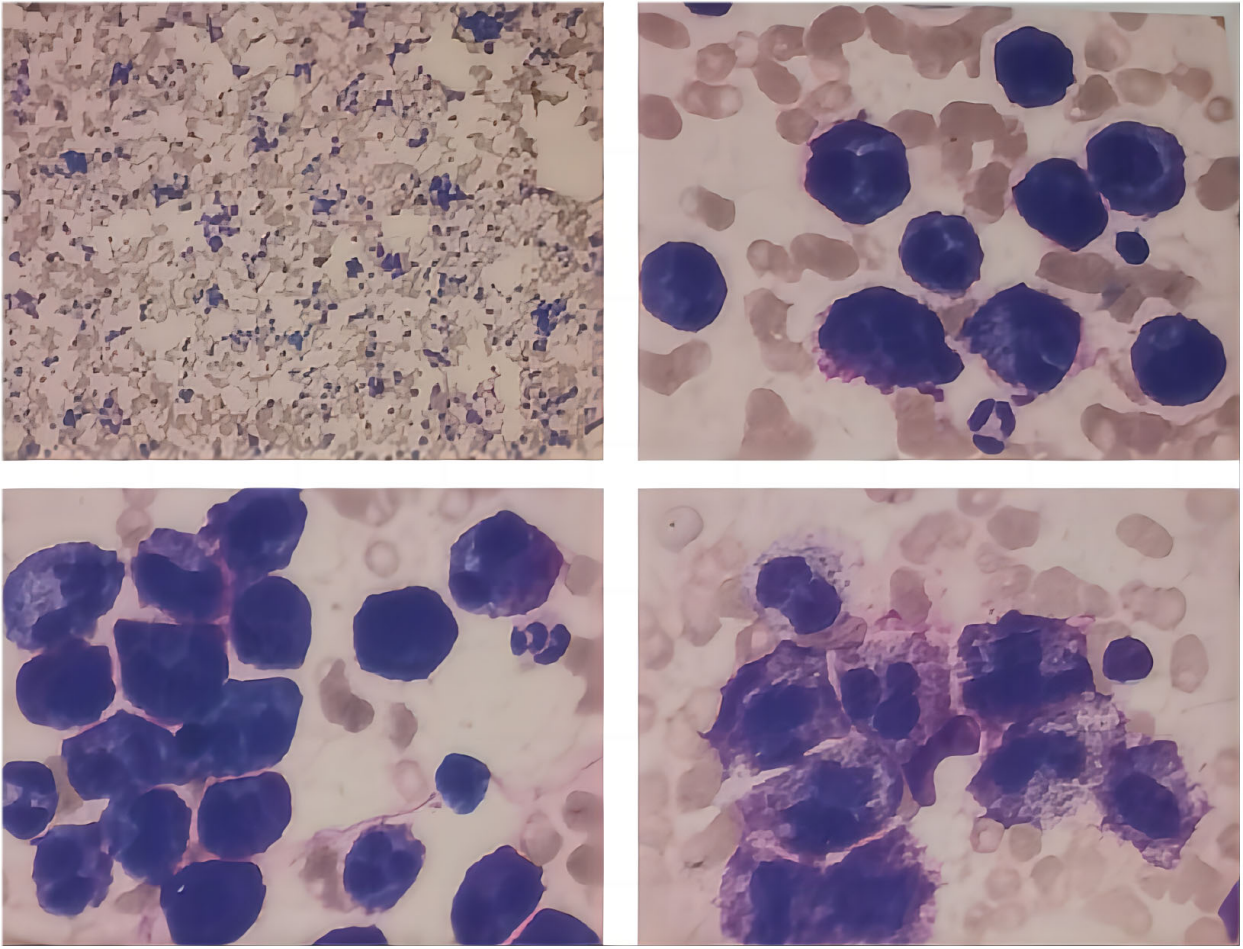
Morphological analysis of bone marrow.

Bone marrow analysis (**Figure 1**) revealed marked hyperplasia, with mast cells constituting 75% of the nucleated cells. These cells were large, with round, oval, or dysmorphic nuclei, often multinucleated, displaying coarse, loose chromatin and abundant cytoplasm containing numerous purple-red granules. The granulocytic series showed a reduced proportion at various stages but appeared morphologically normal. Late-stage normoblasts in the erythroid series were decreased, while other stages exhibited normal morphology. Anisocytosis was noted in red blood cells, including the presence of macrocytes. Lymphocytes were reduced, but otherwise, they appeared normal. Monocytes, megakaryocytes, and platelets appeared normal. No abnormal cells or parasites were detected.

#### 2.2.3 Bone marrow histopathology

Immunohistochemistry: CD3 (focal+), CD20 (focal+), CD71 (minor+), CD61 (±), CD34 (focal+), MPO (focal+), CD138 (focal+).

#### 2.2.4 Flow cytometry

**Figure 2:**
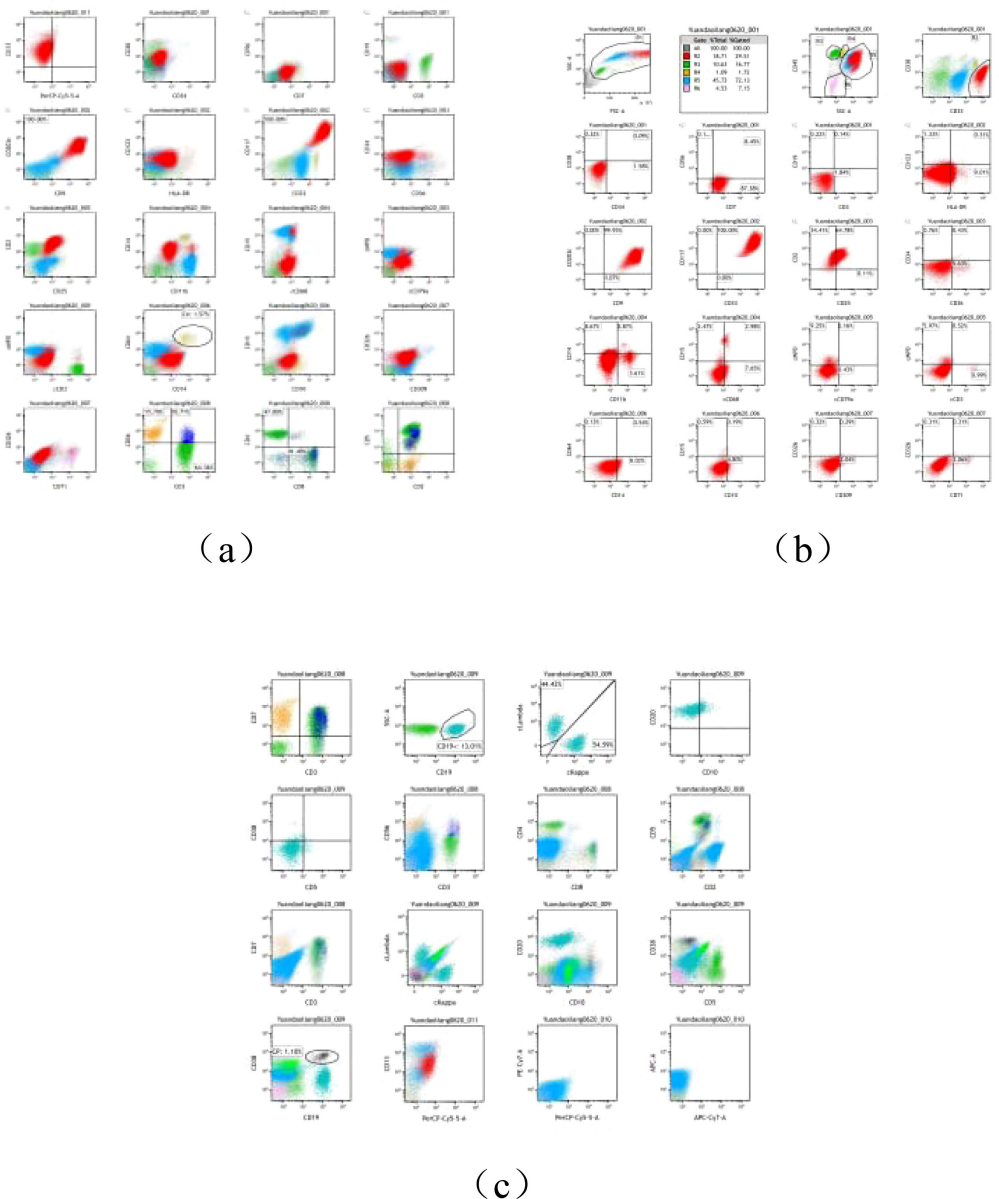
**(A–C)** Flow cytology report.

Bone marrow flow cytometry (**Figure 2**) identified 29.51% of abnormal myeloid cells, some of which expressed CD25. The immunophenotype was consistent with systemic mastocytosis, suggesting mast cell leukemia.

#### 2.2.5 Cytogenetic examination

**Figure 3:**
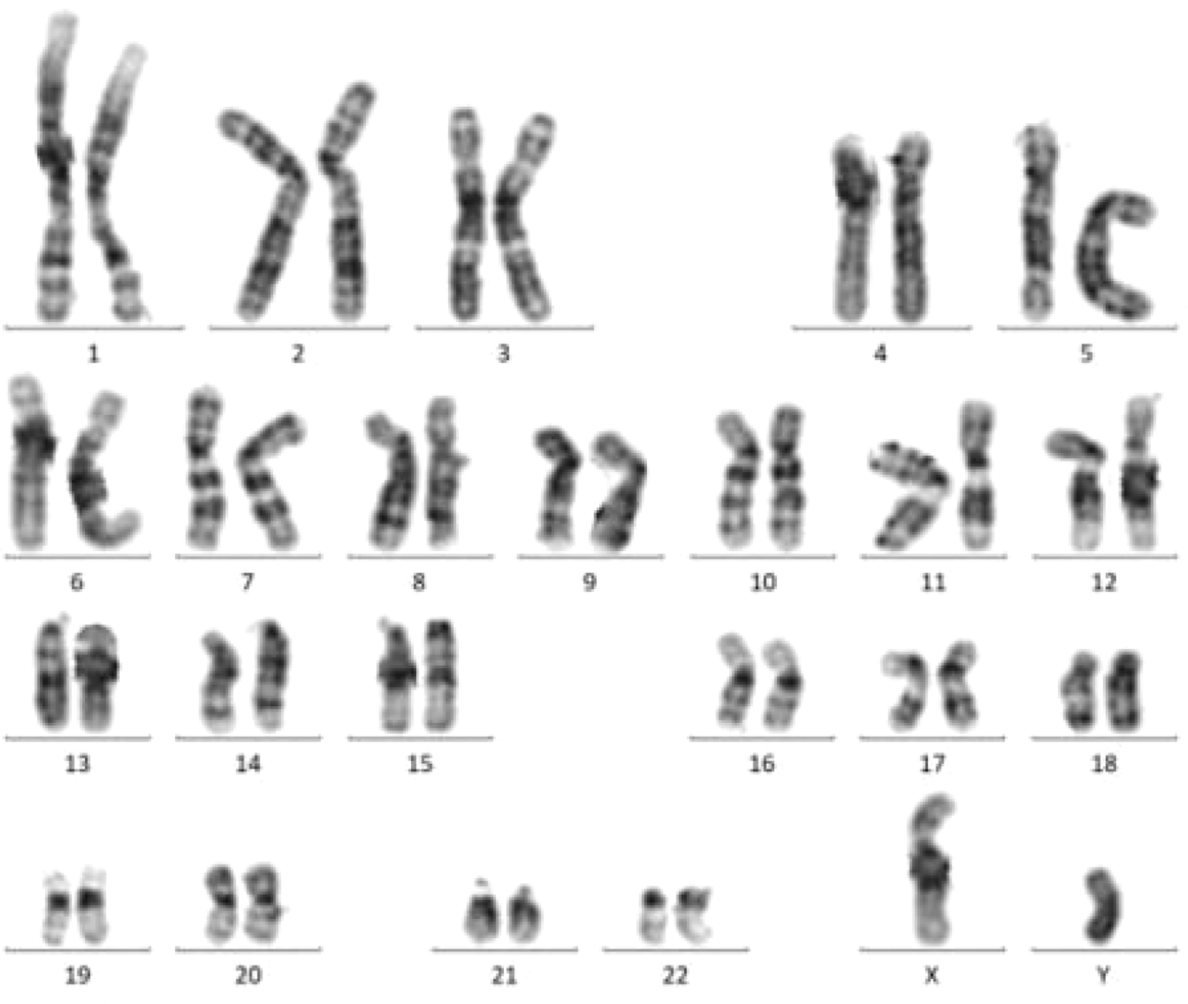
Bone marrow chromosome karyotyping.

Bone Marrow Karyotype (**Figure 3**): No abnormalities were detected. The karyotype was 46, XY【20】.

Myeloid Malignancy-Related Gene Mutation Screening: A panel of 67 genes associated with the diagnosis, treatment, prognosis, and recurrence of myeloid malignancies (including KIT, SRSF2, TET2, and ASXL2) was analyzed, with no relevant mutations identified.

#### 2.2.6 Imaging studies

**Figure 4:**
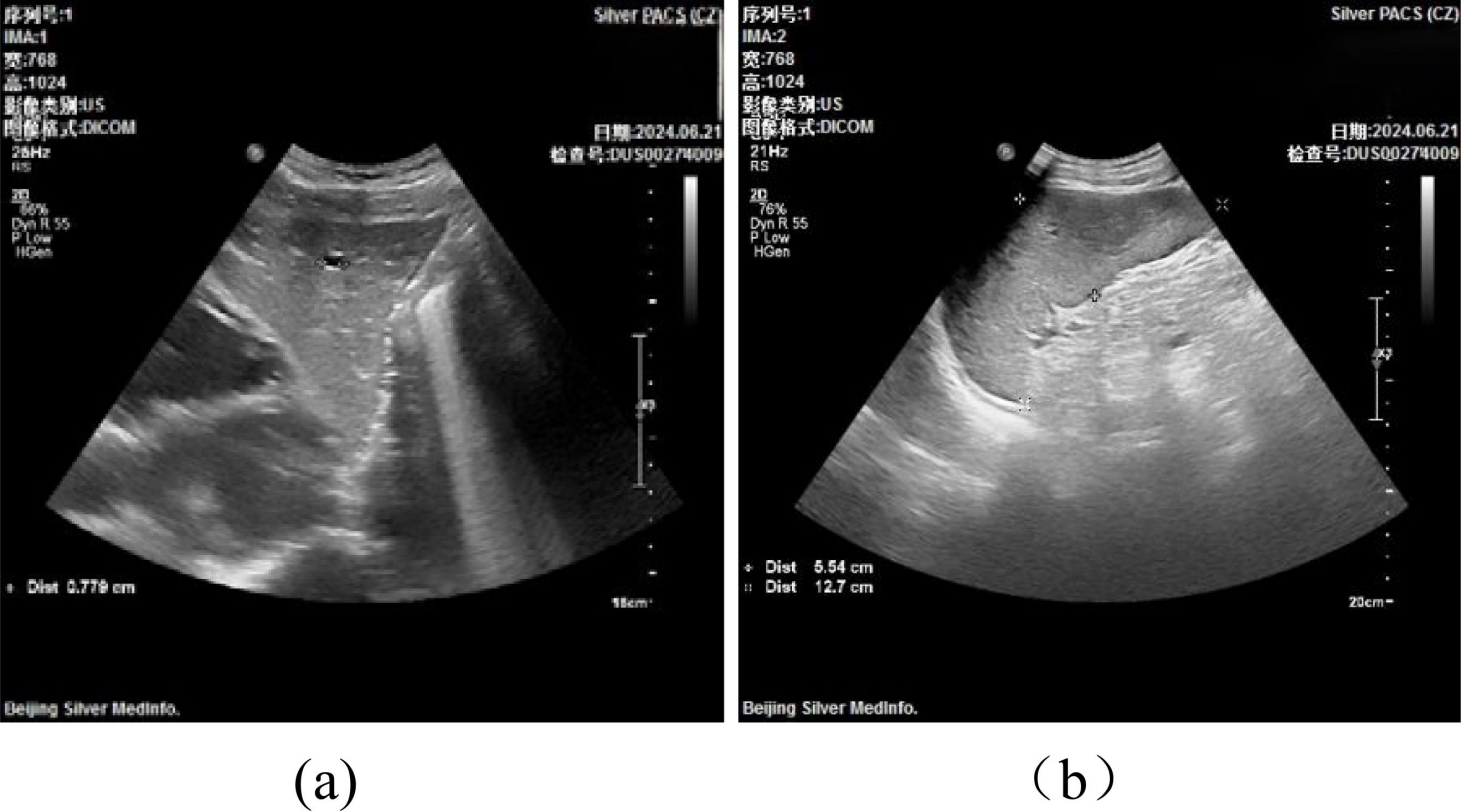
Liver and Spleen Ultrasound.

Liver and Spleen Ultrasound (**Figure 4**): The ultrasound examination of the liver revealed a normal size and shape, although there was a mild increase in the coarse appearance of the parenchymal echotexture. In contrast, the spleen exhibited mild splenomegaly, with a thickness of 5.5 cm and a length of 12.7 cm. The parenchymal echotexture of the spleen was moderately heterogeneous, which further supported the finding of splenomegaly.

### 2.3 Diagnosis

The diagnosis of MCL follows the 2022 WHO diagnostic criteria for SM (3, 6), which require fulfillment of either one major and one minor criterion or at least three minor criteria. In this case, the patient satisfied the major criterion: the presence of multifocal, dense aggregates of mast cells (MCs) in the bone marrow (BM) biopsy or in any extracutaneous organ, with each aggregate containing 15 or more MCs. As for the minor criteria, the patient exhibited more than 25% spindle-shaped, atypical, or immature MCs in either BM biopsy, BM smear, or other extracutaneous organs. Furthermore, MCs expressing CD25, with or without CD2, were present in the patient’s samples. CD25 is considered a more sensitive marker for mast cells compared to CD2, which is less consistent in SM and may decrease as the disease progresses (7). Additionally, the patient also met the criterion of having at least 20% immature or atypical MCs in the BM smear, which further supports the diagnosis of MCL. Thus, the diagnosis of MCL within the context of SM is confirmed.

MCL can be categorized based on its clinical presentation and diagnostic characteristics into primary or secondary forms, as well as acute types with C-findings or chronic types without these findings. Additionally, MCL may occur with or without associated hematologic neoplasms. In this case, the patient exhibited symptoms such as fever, fatigue, weight loss, and diarrhea, accompanied by splenomegaly on ultrasound and laboratory results indicating anemia and thrombocytopenia, confirming the presence of C-findings. These findings align with the study by Kennedy et al. (1), which showed that most patients (86%) had acute MCL and at least one C-finding. However, imaging to assess skeletal involvement was not conducted due to the patient’s decision to decline this examination.

### 2.4 Treatment

Upon admission, the patient presented with a hemoglobin level of 57 g/L. Immediate intervention involved the transfusion of 2 units of leukocyte-depleted, type AB Rh(+) red blood cells. A follow-up blood count performed the day after the transfusion showed an increase in hemoglobin to 77 g/L, confirming the efficacy of the transfusion. After establishing the diagnosis of MCL, symptomatic management was initiated. Literature suggests that antihistamines can help alleviate symptoms arising from mast cell mediator release, while high-dose corticosteroids have been shown to be effective in reducing flushing, nausea, vomiting, abdominal pain, and diarrhea. For this patient, symptom management included the administration of cetirizine hydrochloride tablets (10 mg once daily) and methylprednisolone tablets (8 mg once daily). After two weeks of treatment, the patient reported improvement in diarrhea, with stool consistency returning to normal, and a reduction in pruritus.

Despite the absence of KIT gene mutations, the presence of C-findings underscored the significance of considering mast cell cytoreductive treatments, such as hydroxyurea, α-interferon, cladribine, and allogeneic hematopoietic stem cell transplantation. These therapeutic options were suggested to the patient. However, due to financial limitations, the patient opted not to proceed with these treatments. Following supportive and symptomatic care, there was notable improvement in the patient’s fatigue and pruritus. The patient was subsequently discharged and advised to continue regular follow-up at a local hospital. As of January 2025, the patient’s condition remains stable. He continues to experience some fatigue and occasional fever, but these symptoms have been manageable with self-administered symptomatic treatment. The patient has expressed his intention to continue symptomatic management alone.

## 3 Discussion

MCL is an exceptionally rare condition, with early clinical symptoms that are often nonspecific, complicating the diagnosis and frequently resulting in either misdiagnosis or delayed diagnosis. The overall prognosis of MCL is typically unfavorable. Upon the patient’s initial presentation, symptoms such as fatigue, fever, anemia, diarrhea, pruritus, and weight loss were observed, in addition to splenomegaly on physical examination. Initially, investigations concentrated on common causes of anemia and fever. After excluding infections, autoimmune disorders, nutritional deficiencies, and other potential diseases, no clear diagnostic indications emerged. As a result, we conducted a more detailed history and comprehensive physical examination. Due to the limitations of our hospital, which lacks the capability to measure serum tryptase levels, we performed peripheral blood cytomorphometric analysis and bone marrow aspiration in an attempt to clarify the disease’s etiology. The diagnosis of MCL was confirmed based on the findings from these tests.

Clinical Considerations: The case highlights the importance of comprehensive history-taking and a thorough physical examination in identifying the underlying cause, assessing the extent of tumor involvement, and evaluating organ involvement. A detailed history of mast cell activation symptoms should be meticulously reviewed, with particular attention given to skin manifestations of mastocytosis, allergic conditions, lymphadenopathy, musculoskeletal discomfort, and systemic symptoms such as fatigue, fever, pruritus, weight loss, sweating, and pain induced by alcohol. Additionally, MCL frequently harbors the KIT D816V mutation, and research by Kennedy et al. (1) has identified common co-mutations in genes like SRSF2, TET2, ASXL2, RUNX1, and K/N-RAS. Among these, mutations in SRSF2, ASXL2, and RUNX1 are linked to poorer prognoses. In this case, although the patient exhibited C-findings, he did not receive mast cell attenuation therapy or targeted treatments, aside from symptomatic supportive care, even as he approached one year since the diagnosis of MCL. Follow-up assessments revealed that the patient remained in stable health, with regular check-ups at a local hospital and no acute episodes or hospitalizations. We propose that this stability could be attributed to the absence of mutations in genes associated with poor prognosis, such as SRSF2, ASXL2, and RUNX1, in this patient.

Treatment for MCL typically includes antihistamines to manage symptoms and various supportive therapies. Additionally, mast cell cytoreduction and inhibition of KIT mutations are essential for effective management. Tyrosine kinase inhibitors (TKIs) such as imatinib, dasatinib, and nilotinib, along with multi-target TKIs like midostaurin, have demonstrated clinical efficacy in MCL treatment. Allogeneic hematopoietic stem cell transplantation has also shown potential benefits (7–10). According to a study by Gotlib et al. (11), systemic therapy with midostaurin or avapritinib generally yielded better outcomes compared to cladribine in most MCL patients. Midostaurin has been found effective in roughly two-thirds of patients with advanced SM, significantly reducing bone marrow burden and mitigating organ dysfunction, including cytopenias and liver damage. Although the effects of midostaurin typically last 18–24 months, it is associated with side effects such as nausea, vomiting, and diarrhea. For patients with platelet counts below 50,000/μL, the risk of intracranial hemorrhage increases, making the use of avapritinib contraindicated. Clinical trials are ongoing to explore additional treatment options for MCL. A phase II trial (2018–2026) is specifically evaluating the safety and effectiveness of avapritinib in advanced SM patients. Although allogeneic hematopoietic stem cell transplantation shows promise for MCL, its success largely depends on factors such as the patient’s age, overall health, and the high risk associated with the procedure (12).

Advancements in clinical research have led to increased attention from public health systems toward the management of MCL. In 2017, midostaurin was granted approval by both the FDA and EMA for the treatment of MCL (5). Four years later, in 2021, the FDA approved avapritinib as a first-line therapeutic option for advanced SM, and in 2022, the EMA also endorsed its use as a second-line treatment for MCL (9). In 2024, Canadian health authorities recommended the inclusion of avapritinib in public reimbursement programs for the treatment of MCL, further reflecting the growing recognition of its therapeutic benefits (13).

## 4 Conclusion

This case underscores the complexities associated with diagnosing and treating MCL. The rarity of MCL and its often nonspecific early symptoms contribute to the difficulty in accurately determining its incidence, with comprehensive reviews of its clinical features being scarce. Consequently, a well-established understanding of its pathogenesis and standardized, evidence-based therapeutic guidelines is still lacking. Most of the current insights into the molecular characteristics, treatment strategies, and prognosis of MCL are based on a limited number of case reports and retrospective studies.

While some progress has been made through retrospective analyses, there remain significant gaps in the available knowledge. To address this, it is crucial to enhance multi-center clinical collaborations to establish comprehensive registries for rare diseases like MCL. Gathering and consolidating more real-world data from various sources would increase the sample pool available for research. Furthermore, future studies should not only focus on short-term outcomes but also incorporate extended clinical follow-ups when possible. Ongoing research, international cooperation, and advances in targeted therapies will be vital for improving outcomes in this rare and aggressive form of leukemia.

## Data Availability

The original contributions presented in the study are included in the article/supplementary material. Further inquiries can be directed to the corresponding author.
